# Synthetic Study on the Relationship Between Structure and Sweet Taste Properties of Steviol Glycosides

**DOI:** 10.3390/molecules17044186

**Published:** 2012-04-05

**Authors:** Mani Upreti, Grant Dubois, Indra Prakash

**Affiliations:** Organic Chemistry Department, Global Research and Development, the Coca-Cola Company, One Coca-Cola Plaza, Atlanta, GA 30313, USA; Email: gdubois@coca-cola.com (G.D.); iprakash@coca-cola.com (I.P.)

**Keywords:** reb A, stevioside, steviol, rebAketone, steviosideketone and steviol nor ketone, methylenation

## Abstract

The structure activity relationship between the C_16_-C_17_ methylene double bond on the aglycone of steviol glycosides and the corresponding impact on their sweet taste has been reported here for the first time. It has been observed that converting stevioside and rebaudioside A to their corresponding ketones by switching the doubly bonded methylene on C-17 for a ketone group actually removes the sweet taste properties of these molecules completely. Regenerating the original molecules tends to restore the sweet taste of both the steviol glycosides. Thus this C_16_-C_17_ methylene double bond in rebaudioside A and stevioside can be regarded as a pharmacophore essential for the sweetness property of these molecules.

## 1. Introduction

**Figure 1:**
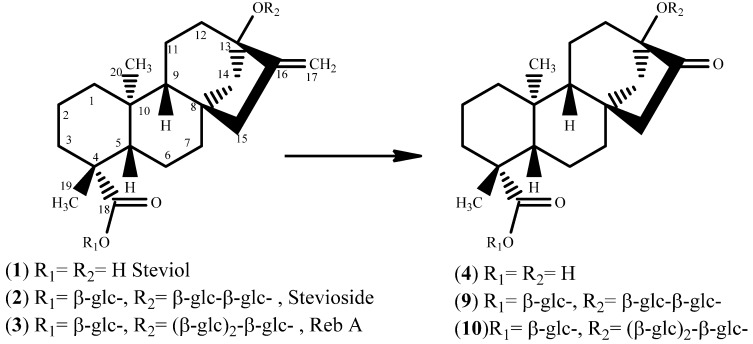
Ketone derivatives of steviol and steviol glycosides.

Rebaudioside A (reb A) and stevioside (Figure 1) are sweet ent-kaurene-type diterpenoid glycosides found in high concentration levels in the leaves of *Stevia rebaudiana*. Steviol (Figure 1) is the aglycone diterpene, while reb A and stevioside are glycosides based on this aglycone’s carbon skeleton. Both stevioside and reb A have a D-glucose group affixed at C_19_ and in addition stevioside has a di-glucosyl, while reb A has a tri-glucosyl sugar moiety affixed at C_13_. Steviol is not sweet at all and it is known that with increasing number of attached glucose moieties the sweetness potency of steviol glycosides tends to increase.

However it is not clear which part of these steviol glycoside molecules is essential for the sweetness properties. The concentration/response (C/R) function of stevioside and reb A has been reported by Dubois *et al*. in 1991 [1]. Based on the C/R functions, R = 10.0C/(200 + C) for reb A and R = 9.9C/(410 + C) for stevioside, the potency (against 5% sugar solution) has been reported to be 120 for stevioside and 250 for reb A [1,2]. The in-house data in Table 1 for reb A in water at 4-6 °C also complies with this reported C/R function:

**Table 1:** Sweetness potency data of reb A.

R (% Sugar Equivalence)	C (Concentration)	Potency (w/w sugar *vs.* reb A)
1.0	26.51	377
2.0	58.23	343
3.0	96.85	310
4.0	145	276
5.0	206.25	242
6.0	287.42	209
7.0	399.81	175
8.0	566	141

Due to our interest in the chemistry of these steviol glycosides, we have been investigating various ways of modifying these natural molecules in order to find relations between various parts of their structures and the sweet taste properties. We have found that converting stevioside and reb A into their corresponding ketones by switching the doubly bonded methylene on the C_17_ with a ketone actually removes the sweet taste properties of these molecules completely. Interestingly, upon regenerating the original molecules back, the sweet taste of both the steviol glycosides were found to be restored. This finding indicates that the C_16_-C_17_ parts of reb A and stevioside are crucial for the sweetness properties of these molecules. We describe here in detail the various chemical manipulations we carried have out to arrive at these findings.

## 2. Results and Discussion

Preparation of steviol ketone has previously been reported in the literature [3]. This *Phytochemistry* article has described use of radiolabeled steviol for the synthesis of radiolabeled gibberellins using the fungus *Gibberella Fujikuroi*. In this article the labeled steviol was synthesized from steviol acetate nor-ketone by the Wittig reaction using [^14^C](methyl)triphenyl phosphonium iodide. However there are no other similar literature reports available for similar synthetic transformations on reb A or stevioside molecules.

We first repeated the literature reported Wittig methylenation reaction procedure on steviol acetate ketone to see if it worked in our hands, but we were not very successful with this procedure. Further Wittig reaction was also tried on reb A peracetate nor-ketone and stevioside peracetate nor-ketone. The reaction conditions seemed to deacetylate the starting molecules and the resulting reaction mixtures seemed to be mixtures of several products. Apparently this reaction did not seem to work for any of these molecules.

**Scheme 1:**
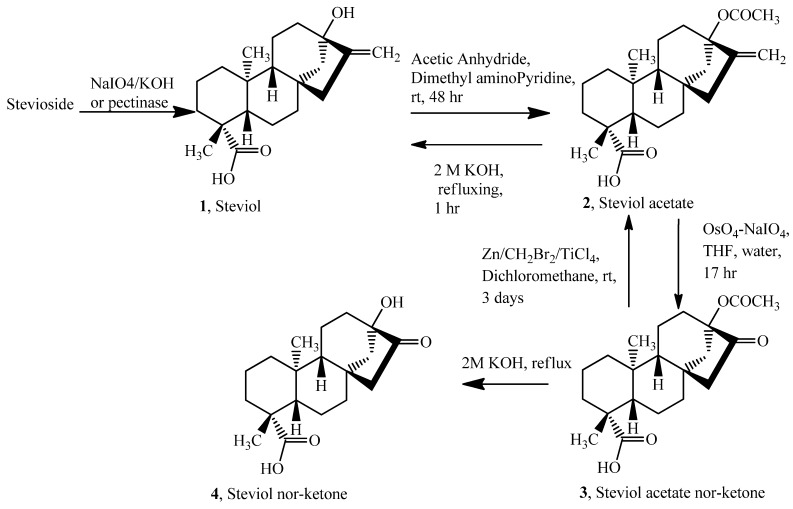
From stevioside to steviol acetate nor-ketone and steviol re-generation.

**Scheme 2:**
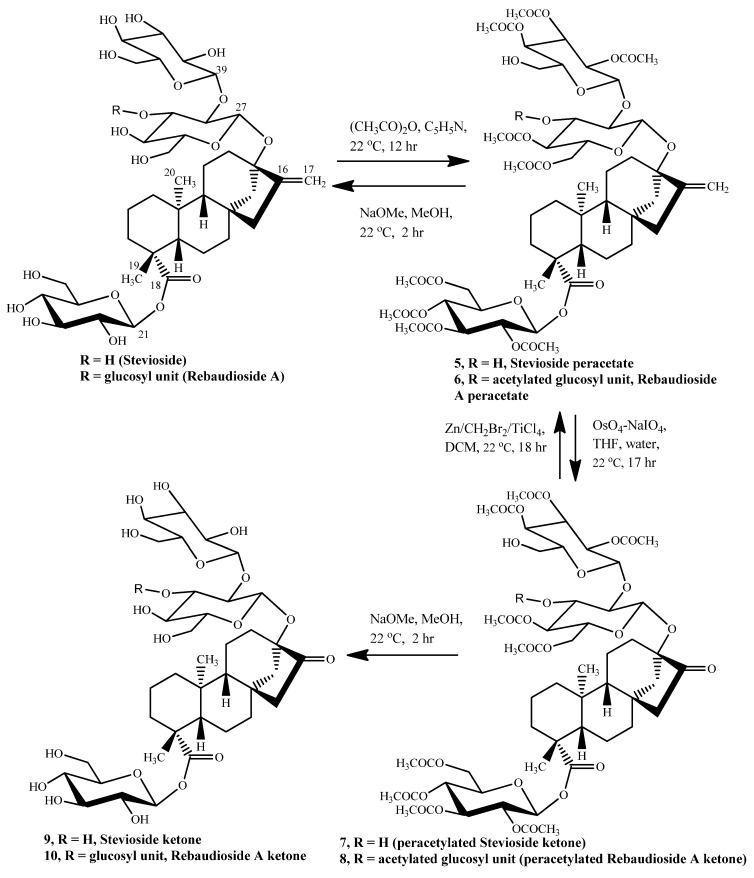
The synthetic transformations of stevioside and rebaudioside A molecules.

We therefore designed a four step novel approach (Scheme 1 and Scheme 2) taking advantage of the presence of the similar diterpene aglycone in all three of these compounds: Steviol, stevioside and reb A. Briefly the starting molecule was first acetylated and then was oxidized at the aglycone part to the 17-nor-16-ketone. This intermediate ketone was then converted back to C_17_ methylene form using TiCl_4_-Zn-CH_2_Br_2_ reagent [4] in dry THF as solvent, (Lombardo’s procedure) which is a mild non-basic method for the methylenation of ketones.

The methylenation reaction required anhydrous conditions, therefore reb A and stevioside diterpenoid glycosides and steviol were first converted to their non-polar per-acetate forms. For this purpose hydroxy protection methods such as acetylation and silylation were tried. Silylation did not seem to work well as in case of reb A it resulted in an oily substance which was hard to dry and did not give the right molecular weight expected for the desired per-silylated reb A. Instead it was a mixture of partially silylated reb A derivatives. Acetylation under traditional pyridine-acetic anhydride conditions seemed to work well for both reb A and stevioside and was a quantitative reaction. Steviol acetate however was prepared in presence of dimethyl amino pyridine and triethylamine in order to acetylate the tertiary hydroxy group [5]. Hydroxyl functionalities thus protected with base labile acetyl groups survived the ketone formation conditions and further methylenation step and de-protection were carried out by basic hydrolysis under sodium methoxide-methanol conditions.

To prepare the intermediate ketones, the most obvious method seemed to be ozonolysis. However this seemingly common reaction could not be optimized for these diterpenoid molecules as it did not give consistent results under the present laboratory conditions. At the same time the oxidation reaction using osmium tetroxide and sodium periodate was attempted and it worked for all the three molecules. This method always resulted in per-acetylated ketone, which is the desired reaction product, along with unreacted starting material. It was difficult to resolve reb A and stevioside peracetate nor-ketones on silica-gel column/TLC by any variation in the solvent systems, hence extensive purification was followed here to get pure intermediate ketones out of these oxidation reactions. Scaling-up of the ketone formation reaction or increase in equimolar amounts of osmium tetroxide and sodium periodate did not result in any enhanced ketone yields as well. 

The intermediates, stevioside and reb A per-acetate and its ketones prepared here are novel compounds being reported for the first time and similarly TiCl_4_-Zn-CH_2_Br_2_ reagent’s methylenation reaction with steviol acetate nor-ketone, stevioside and reb A per-acetate ketones are also the first such reports in the literature. During analytical characterization of these novel ketone derivatives, we found that in the carbon NMR spectra, the carbonyl carbon of steviol and reb A ketones tends to appear downfield around δ 214–215 while in stevioside ketone, it appears a little upfield around δ 192. This difference in signal values tells that the carbonyl carbon at C-16 is deshielded to a different degree in reb A and steviol as compared to stevioside. The stevioside and reb A ketone derivatives in purified form and their regenerated stevioside and reb A were taste tested by authors (Dubois and Prakash) who are also in-house sensory experts following an in-house developed “sip and spit protocol” at 100 ppm concentrations. It was concluded that ketone formation thus led to absence of sweet taste for corresponding stevioside and reb A derivatives.

## 3. Experimental Section

### 3.1. General

The starting materials used for steviol and stevioside work were not readily available. Preparation of the starting material steviol was carried out in-house by slightly modifying a chemical method reported in a Japanese patent [6] involving sodium periodate treatment followed by alkaline hydrolysis and by an enzymatic method by hydrolysis of stevioside with crude pectinase from *Aspergillus Niger*. Both methods gave satisfactory results upon comparing with a commercial sample of steviol from Sigma-Aldrich. The starting material for stevioside work was a crude stevioside mixture containing only 55% stevioside. Pure stevioside peracetate was purified from this mixture after the acetylation reaction. The +99% pure reb A were obtained from in-house crystallization work. NMR spectra were acquired on Varian Unity Plus 600 MHz instruments using standard pulse sequences. The chemical shifts (δ) are expressed in ppm in relation to tetramethylsilane as a standard and the coupling constants (*J*) are given in Hz. MS data were generated with a Waters Premier Quadrupole Time-of-Flight (Q-Tof) mass spectrometer equipped with an electrospray ionization source operated in the positive-ion and negative-ion mode. Samples were diluted with water: acetonitrile (1:1) containing 0.1% formic acid and introduced via infusion using the onboard syringe pump. HPLC analysis was carried out for steviol and its derivatives via Phenomenex Hydro-RP, 150 × 4.6 mm, eluent A: 10% CH_3_CN-50 mM H_3_PO_4_, eluent B: 100% CH_3_CN, *t_0_* A:B = 100:0, *t_45_* A:B = 0:100, flow 1.0 mL/min, UV 210 nm, and for stevioside, reb A and their derivatives via Zorbax amino column, 4.6 × 150 mm using an isocratic gradient of A = 75% acetonitrile-25% water-0.05% acetic acid and B = 100% acetonitrile-0.05% acetic acid (A:B = 60:40), 1.0 mL per minute using UV detection at 210 nm. 

### 3.2. 4% Aqueous Solution of OsO_4_

A 250 mg capsule of OsO_4_ was broken into a wide mouth glass bottle containing 6.25 mL of water. The bottle was immediately capped and then swirled for 15–20 min in order to dissolve all the OsO_4 _crystals in water. 

### 3.3. Zn/CH_2_Br_2_/TiCl_4_ Reagent

TiCl_4_ (2.3 mL) was added dropwise over 10 min to a stirred suspension of zinc dust (5.75 g) in CH_2_Br_2_ (2 mL) and THF (50 mL, freshly distilled over sodium and benzophenone) at −40 °C. The mixture was allowed to warm to 5 °C and was then stirred at this temperature for 3 days to give a thick grey slurry of an active species. It was stored at −20 °C.

*Steviol* (**1**) - (i) *Chemical Method *[6]: Stevioside (1.0 gm, 1.24 mmol) was suspended in water (75 mL) and treated with sodium periodate (1.5 g, 7.0 mmol) for 21 h at room temperature. The white frothy solution was then freeze-dried and was redissolved in 10% KOH solution (50 mL). The clear solution thus obtained was heated at 85 °C in an oil bath fitted with a reflux condenser. It was cooled and neutralized with 2% aqueous HCl to pH = 5.0. Heavy precipitation appeared at this time. The reaction mixture was then extracted with ethyl acetate three times (30 mL ea.). The combined ethyl acetate solution was dried over sodium sulfate and evaporated under reduced pressure to get 270 mg of crude steviol, which was absorbed on silica and purified by column chromatography with 35–50% ethyl acetate in hexane as eluent. 150 mg of pure steviol was obtained (**1**, yield = 35%). TLC (hexane: ethyl acetate, 1:1): Rf = 0.29; ESI-MS *m/z*: 317.2 [M−H]^−^, Calcd. 317.44 for C_20_H_30_O_3_[M−H]^−^; HPLC (*t*R, 26.85 min).

*Steviol* (**1**) - (*ii) Enzymatic Method *[3]: Stevioside (0.5 g, 0.62 mmol) was hydrolyzed at 45 °C with crude pectinase from *Aspergillus Niger* (1.0 mL, Sigma-Aldrich, P2736) in 0.1 M phosphate buffer (50 mL), pH = 4.5 for 24 h. The crude steviol precipitated out during the reaction. It was filtered and then dissolved in acetonitrile. The acetonitrile layer was left overnight at room temperature when some more material precipitation occurred. It was again filtered and the acetonitrile filtrate was evaporated under reduced pressure. Crude product thus obtained was crystallized from methanol to give white needle like crystals of pure steviol (**1**, yield = 25%).

*Steviol Acetate *(**2**). Steviol (**1**, 0.954 g, 3 mmol), 4-*N,N*-dimethylaminopyridine (96 mg) and acetic anhydride (1.8 mL) were dissolved in anhydrous triethylamine (5 mL) and the reaction mixture was stirred at room temperature for 48 h [5]. To this methanol (2 mL) was added in order to remove the excess of acetic anhydride followed by evaporation on a rotavapor at 30 °C under reduced pressure. The residue was dissolved in ether (20 mL) and washed with water (3 × 8 mL), 2% HCl (3 × 8 mL) followed by washing with dilute sodium bicarbonate (5%, 3 × 8 mL) and one final wash with brine (8 mL). The ether layer was dried over sodium sulfate and evaporated on a rotavpor at 30 °C under reduced pressure to give crude steviol acetate. Recrystallization by acetone-water (5:1) gave 0.380 gm of white needle like crystals of pure steviol acetate (56%). TLC (hexane: ethyl acetate, 1:1): Rf = 0.68; ^1^H-NMR (CDCl_3_) δ = 4.905 (s, 1H, H-17), 4.880 (s, 1H, H-17), 2.034 (s, 3H, OCOCH_3_*)*, 1.261 (s, 3H, H-19) 1.01(s, 3H, H-20); ^13^C-NMR: δ = 152.679 (C=CH_2_, C-16), 103.640 (C=CH_2_, C-17); ESI-MS *m/z*: 383.4 [M+Na]^+^, Calcd. 383.48 for C_22_H_32_O_4_[M+Na]^ +^; HPLC (*t*R, 34.8 min).

*Steviol Acetate Ketone* (**3**) [3]. Steviol acetate (**2**, 320 mg, 0.88 mmol) was dissolved in a mixture of THF (3.2 ml) and water (2.8 mL). To this was added freshly made 4% aqueous solution of OsO_4_ (0.4 mL). After 10 min the reaction mixture had become deep brown and to this NaIO_4_ (672 mg) was added slowly over a period of 2–3 min and the mixture was left for stirring at room temperature for 18 h. It had become a thick white suspension by now and was extracted with ethyl acetate (3 × 30 mL). The organic layer was dried over sodium sulfate and evaporated on a rotavapor at 30 °C under reduced pressure to get crude steviol acetate ketone, which was flash chromatographed on silica gel 60 (70–230 mesh) with a stepwise gradient from 10% ethyl acetate in hexane to 40% ethyl acetate in hexane. Pure steviol acetate ketone came out in fractions collected at 35% ethyl acetate in hexane, yield = 230 mg (71%). TLC (hexane: ethyl acetate, 1:1): Rf = 0.41; ^1^H-NMR (CDCl_3_) δ = 2.069 (s, 3H, OCOCH_3_), 1.273 (s, 3H, H-19) 1.038(s, 3H, H-20); ^13^C-NMR: δ = 214.106 (C=O, C-16), peaks at 152.679 and 103.640 present in **2** disappear here; ESI-MS *m/z*: 385.4 [M+Na]^+^, Calcd. 385.45 for C_21_H_30_O_5_ [M+Na]^+^, HPLC(*t*R, 25 min).

*Methylenation of Steviol Acetate Ketone* [4]. Steviol acetate ketone (**3**, 100 mg, 0.27 mmol) was taken up in anhydrous dichloromethane (4 mL) and to this solution was slowly added Zn/CH_2_Br_2_/TiCl_4 _reagent (5 mL, 7.1 equivalents, cold reagent). The reaction mixture was stirred for five days at room temperature and then quenched by pouring into a biphasic 2:1 mixture of sodium bicarbonate-water (10 mL) and ether (10 mL). The aqueous layer was extracted two more times with ether (10 mL ea.). The combined ether layer was dried over sodium sulfate and evaporated on a rotavapor at 30 °C under reduced pressure to afford a crude product that was chromatographed on silica gel 60 (70–230 mesh) with a stepwise gradient from 5% ethyl acetate in hexane to 20% ethyl acetate in hexane. First an impurity eluted at 15% ethyl acetate/hexane and after this at 20% ethyl acetate /hexane came a product similar to steviol acetate (**2**) on TLC, yield = 60 mg (60%). TLC (hexane-ethyl acetate, 1:1): Rf = 0.68; ^1^H-NMR (CDCl_3_) δ = 4.916 (s, 1H, H-17), 4.876 (s, 1H, H-17), 2.060 (s, 3H, OCOCH_3_), 1.292 (s, 3H, H-19), 1.001(s, 3H, H-20);^ 13^C-NMR: δ = 152.604 (C=CH_2_, C-16), 103.867 (C=CH_2_, C-17);. ESI-MS *m/z*: 383.4 [M+Na]^+^, Calcd. 383.48 for C_22_H_32_O_4_[M+Na]^+^, HPLC (*t*R, 34.8 min).

*Steviol nor-ketone *(**4**). Steviol acetate nor-ketone (40 mg, 0.11 mmol) was refluxed in 2 M KOH (20 mL) for one hour and then cooled. The cooled reaction mixture was then acidified with 30% HCl to pH = 2.5 whereupon the solution becomes milky and a white precipitate appears. It was extracted with ethyl acetate (3 × 15 mL), dried over sodium sulfate and then evaporated on a rotavapor at 30 °C under reduced pressure, yield = 30 mg, 85%. TLC (hexane-ethyl acetate, 1:1): Rf = 0.28; ^1^H-NMR (CDCl_3_) δ = 1.205 (s, 3H, H-19), 0.940 (s, 3H, H-20); ^13^C-NMR: δ = 214.201 (C=O, C-16); ESI-MS *m/z*: 319.4 [M−H]^−^, Calcd. 319.42 for C_19_H_28_O_4_[M−H]^−^, HPLC (*t*R, 27 min). A similar procedure was followed for de-acetylation of **2** to **1**. 

*Stevioside* *peracetate* (**5**). In a 500 mL round bottom flask, stevioside (55% stevioside by HPLC analysis, 15 g, nearly 10.25 mmol) was dried overnight in a vacuum oven at 75 °C. To this was added dry pyridine (200 mL) followed by cooling to 0 °C and then addition of acetic anhydride (350 mL). It was allowed to warm up to room temperature and then stirred at room temperature for 48 h. The reaction was then poured on a beaker containing ice and shaken vigorously. It was then extracted with diethyl ether (3 × 50 mL) followed by washing the organic layer with cold water (3 × 30 mL), 5% hydrochloric acid (3 × 30 mL), cold saturated sodium bicarbonate till no further evolution of CO_2_ was seen (3 × 40 mL) and then finally by brine (30 mL). The organic layer was dried over sodium sulfate and then evaporated to get the crude per-acetylated stevioside which was purified by silica gel flash chromatography (230–400 mesh) using a stepwise gradient of dichloromethane-methanol from pure dichloromethane up to 10% methanol in dichloromethane, yield = 10.00 g (77%). TLC (dichloromethane-methanol, 10:0.5): Rf = 0.52;^ 1^H-NMR (DMSO) δ = 6.016 (d, 1H, H-21, *J *= 8.4 Hz); ^13^C NMR δ = 153.016 (C=CH_2_, C-16), 103.792 (C=CH_2_, C-17); ESI-MS *m/z*: 1289.8 [M+Na]^+^, Calcd. 1290.27 for C_60_H_82_O_29_ [M+Na]^+^.

*Stevioside per acetate Ketone *(**7**). Stevioside per-acetate (**5**, 866 mg, 1.1 mmol) was taken up in a mixture of THF (4.0 ml) and water (3.5 mL). To this was added freshly made 4% aqueous solution of OsO_4_ (0.5 mL). After 10 min the reaction mixture had become deep brown and to this NaIO_4_ (840 mg) was added slowly over a period of 2–3 min and the mixture was left for stirring at room temperature for 17 h. It had become a thick white suspension by now and was extracted with ethyl acetate (2 × 40 mL). The organic layer was dried over sodium sulfate and evaporated on arotavapor at 30 °C under reduced pressure to give the crude stevioside per-acetate ketone, which was chromatographed on silica gel 60 (70–230 mesh) with a stepwise gradient from pure dichloromethane to 2.5% methanol in dichloromethane. Pure ketone started coming out in fractions collected at 2% dichloromethane in methanol, yield = 378 mg (27%). TLC (DCM–MeOH, 10:0.5): Rf = 0.42; ^1^H-NMR (DMSO) δ = 6.024 (d, 1H, H-21, *J *= 8.4 Hz); ^13^C-NMR δ = 191.228 (C=O, C-16), peaks at δ 153.016 & 103.792 present in **5** disappear here; ESI-MS *m/z*: 1292.0 [M+Na]^+^, Calcd. 1292.24 for C_59_H_80_O_30_ [M+Na]^ +^.

*Methylenation of stevioside acetate ketone and re-generation of stevioside*. Stevioside peracetate ketone (500 mg, 0.393 mmol) was taken up in anhydrous dichloromethane (10.5 mL) and to this solution was slowly added, under nitrogen, Zn/CH_2_Br_2_/TiCl_4 _reagent (10 mL, 8.3 equivalents, cold reagent). The reaction mixture was stirred for 18 h. at room temperature when the TLC in DCM–MeOH (20:1) showed disappearance of ketone. The reaction was further monitored by HPLC on an amino column. On the basis of HPLC and LC-MS of t = 0 and t = 18 h. samples, the reaction appeared complete. It was then quenched by pouring into a biphasic mixture of saturated sodium bicarbonate (20 mL) and ethyl acetate (10 mL). The aqueous layer was extracted two more times with ethyl acetate (10 mL ea.). The combined ethyl acetate layer was dried over sodium sulfate and evaporated on a rotavapor at 30 °C under reduced pressure to afford a crude product that was taken up in anhydrous methanol (10 mL). To this was added anhydrous sodium methoxide (200 mg). It was stirred at room temperature for 2 h and then neutralized by 0.5% HCl to pH ~ 6.5–7.0. The methanol was removed on a rotavapor under reduced pressure. A 1.0 mL size short column of reverse phase silica, C18, was packed in 50% acetonitrile-water and then washed with 50–100 mL double distilled water. The de-acetylated product was dissolved in minimum amount of water and loaded on this C18 column followed by washing with water (100 mL). Elution with 90% acetonitrile-water (50 mL) resulted in pure stevioside, as shown by HPLC (*t*R, 3.51, same as standard stevioside). The acetonitrile solution was evaporated and dried to get pure stevioside, yield = 208 mg (68%).

*Stevioside ketone *(**9**). Stevioside peracetate ketone (500 mg, 0.393 mmol) was taken up in anhydrous methanol (10 mL). To this was added anhydrous sodium methoxide (200 mg). It was stirred at room temperature for 2 h. and then neutralized by 0.5% HCl to pH ~ 6.5–7.0. The methanol was removed on a rotavapor under reduced pressure followed by desalting on a pre-packed C-18 cartridge (500 mg) from Varian. The de-acetylated product was further purified via preparative HPLC on a C-18 column to get pure stevioside ketone, yield = 158 mg (55%). ^1^H-NMR (DMSO) δ = 1.212 (s, 3H, H-19), 0.983 (s, 3H, H-20); ^13^C-NMR: δ = 191.230 (C=O, C-16); ESI-MS *m/z*: 809.1 [M+H]^+^, Calcd. 807.85 for C_37_H_58_O_19_ [M+H]^ +^.

*Per-acetylated reb A* (**6**). In a 500 mL round bottom flask, rebaudioside A (+99% by crystallization, 5 g, 5.17 mmol) was dried overnight in a vacuum oven at 75 ^ο^C. To this was added dry pyridine (150 mL) followed by cooling it to 0 ^ο^C and then addition of acetic anhydride (250 mL). It was allowed to warm up to room temperature and then stirred for 48 h. The reaction was then poured on a beaker containing ice and shaken vigorously. It was then extracted with diethyl ether (3 × 50 mL) followed by washing the organic layer with cold water (3 × 30 mL), 5% hydrochloric acid (3 × 30 mL), cold saturated sodium bicarbonate till no further evolution of CO_2_ was seen (3 × 40 mL) and then finally by brine (30 mL). The organic layer was dried over sodium sulfate and then evaporated to get the per-acetylated rebaudioside A as a single spot on TLC, yield = 6.5 g (80%). TLC (DCM–MeOH, 10:0.5): Rf = 0.36; ^1^H-NMR (DMSO) δ = 5.982 (d, 1H, H-21, *J *= 7.8 Hz); ^13^C-NMR δ = 152.890 (C=CH_2_, C-16), 104.292 (C=CH_2_, C-17); ESI-MS *m/z*: 1578.5 [M+Na]^+^, Calcd. 1578.52 for C_72_H_98_O_37_[M+Na]^+^.

*Rebaudioside A Per-Acetate Ketone* (**8**). Rebaudioside A peracetate (800 mg, 0.51 mmol) was taken up in a mixture of THF (4.0 mL) and water (3.5 mL). To this was added freshly made 4% aqueous solution of OsO4 (0.5 mL). After 15 minthe reaction mixture had become deep brown and to this NaIO_4_ (840 mg) was added slowly over a period of 2–3 min and the mixture was left for stirring at room temperature for 18 h. It had become a thick white suspension by now and was extracted with ethyl acetate (2 × 40 mL). Organic layer was dried over sodium sulfate and evaporated on a rotavapor at 30 °C under reduced pressure to get crude rebaudioside A per-acetate ketone that was chromatographed on silica gel 60 (70–230 mesh) with a slow stepwise gradient from pure dichloromethane to 2.5% methanol in dichloromethane, yield = 220 mg (30%). TLC (DCM–MeOH, 10:0.5): Rf = 0.28; ^1^H-NMR (DMSO) δ = 5.982 (d, 1H, H-21, *J *= 7.8 Hz), ^13^C-NMR δ = 215.749 (C=O, C-16), peaks at δ152.890 and 104.292 present in **6 **disappear here; ESI-MS *m/z*: 1580.0 [M+Na]^+^, Calcd. 1580.5 for C_71_H_96_O_38_[M+Na]^+^.

*Methylenation of Rebaudioside A Acetate Ketone and re-generation of reb A*. Rebaudioside A per- acetate ketone (**8**, 233 mg, 0.149 mmol) was taken up in anhydrous dichloromethane (4 Ml). To this solution was slowly added under nitrogen, Zn/CH_2_Br_2_/TiCl_4 _reagent (4 mL, 8.7 equivalents, cold reagent). The reaction mixture was stirred at room temperature and was monitored by HPLC on an amino column (it was hard to interpret the TLC data due to the appearance of several spots below the actual ketone spot, which were probably the de-acetylation products). A series of aliquots of the reaction mixture were taken out at t = 18, t = 41 and t = 72 h (each ~400 μL, 7 μmol) to monitor the reaction. They were first quenched with sat. sodium bicarbonate and ethyl acetate (1:1) followed by isolation, drying and evaporation of ethyl acetate layer and then de-acetylation. On the basis of HPLC and LC-MS of t = 0 and t = 18, 41, 72 h. samples the reaction seemed complete after 72 h. The reaction was then quenched by pouring into a biphasic mixture of saturated sodium bicarbonate (10 mL) and ethyl acetate (5 mL). The aqueous layer was extracted two more times with ethyl acetate (5 mL ea.). The combined ethyl acetate layer was dried over sodium sulfate and evaporated on a rotavapor at 30 ^ο^C under reduced pressure to get a crude product which was only 37 mg. The crude was taken up in anhydrous methanol (4 mL). To this was added anhydrous sodium methoxide (19 mg). It was stirred at room temperature for 2 h. and then neutralized by 0.5% HCl to pH ~ 6.5–7.0. The methanol was removed on a rotavapor under reduced pressure. A 500 mg pre-packed C-18 cartridge from Varian was first treated with 50% acetonitrile-water and then washed with 15–20 mL double distilled water. The de-acetylated product was dissolved in minimum amount of water and loaded on C-18 column followed by washing with water (15 mL). Elution with 90% acetonitrile-water (15 mL) resulted in pure reb A as shown by HPLC. The acetonitrile solution was evaporated and dried to get pure reb A, yield = 17 mg (14.5% yield).

*Reb A ketone *(**10**). Rebaudioside A peracetate ketone (**8**, 233 mg, 0.149 mmol) was taken up in anhydrous methanol (4 mL). To this was added anhydrous sodium methoxide (19 mg). It was stirred at room temperature for 2 h. and then neutralized by 0.5% HCl to pH ~ 6.5–7.0. The methanol was removed on a rotavapor under reduced pressure followed by desalting on a pre-packed C-18 cartridge (500 mg) from Varian. The de-acetylated product was further purified via preparative HPLC on a C-18 column to get pure reb A ketone, yield = 31 mg (22%). ^1^H-NMR (DMSO) δ = 1.204 (s, 3H, H-19), 0.979 (s, 3H, H-20); ^13^C-NMR: δ = 215.457 (C=O, C-16); ESI-MS *m/z*: 992.1 [M+Na]^+^, Calcd. 991.98 for C_43_H_68_O_24_[M+Na]^+^.

## 4. Conclusions

We have investigated the relationship between the presence or absence of the methylene double bond on the aglycone of steviol glycosides and the corresponding impact on taste. We have found that converting stevioside and reb A to their corresponding ketones by switching the doubly bonded methylene on the C_17_ with a ketone actually removes the sweet taste properties of these molecules completely. Upon regenerating the original molecules, the sweet taste of both the steviol glycosides were found to be restored. These findings indicate that the C_16_-C_17_ part of reb A and stevioside is an essential pharmacophore for the sweetness properties of these molecules. This is also the first literature report of stevioside and reb A peracetate and its novel ketones derivatives. The methylenation reaction of these ketone derivatives using TiCl_4_-Zn-CH_2_Br_2_ reagent is also the first such report in the literature.
